# Apelin involved in progression of diabetic nephropathy by inhibiting autophagy in podocytes

**DOI:** 10.1038/cddis.2017.414

**Published:** 2017-08-24

**Authors:** Yu Liu, Jia Zhang, Yangjia Wang, Xiangjun Zeng

**Affiliations:** 1 Department of Pathology and Pathophysiology, Basic Medical School of Capital Medical University, Beijing 100069, China

## Abstract

Podocyte autophagy dysfunction has been reported to be responsible for the progression of diabetic nephropathy (DN), however, the factors contributed to autophagy dysfunction in type 2 diabetes are not fully understood. Among promoting factors in DN, an adipokine, apelin, had been showed to trigger podocyte dysfunction. Therefore, it is hypothesized that apelin, which is increased in plasma in type 2 diabetes, lead to podocyte apoptosis through inhibiting podocyte autophagy, which resulted in podocyte dysfunction followed by DN. KkAy mice (diabetic mice) and cultured podocytes (MPC5 cells and native podocytes) were treated with high glucose (HG) and apelin or its antagonist F13A. Renal function, podocyte autophagy, podocyte apoptosis and corresponding cell signaling pathways in podocytes were detected. The results showed that apelin aggravated the renal dysfunction and foot process injuries in kkAy mice, which is positively correlated to podocyte apoptosis and negatively correlated to podocyte autophagy. Apelin induced podocyte apoptosis and inhibited podocyte autophagy in both normal glucose and HG conditions while F13A reversed these effects. Investigations by western blot found that apelin inhibits podocyte autophagy through ERK-, Akt- and mTOR-dependent pathways. In conclusion, increased apelin concentration in plasma inhibited podocyte autophagy, which would lead to podocyte apoptosis and renal dysfunction in diabetes. These effects would contribute to the progression of DN.

Podocytes are predominantly responsible for maintaining the glomerular filtration barrier, whose injuries have an important role in the progression of diabetic nephropathy (DN).^1, 2^ Podocytes are highly specialized, terminally differentiated and unable to proliferate, which result in the condition that podocytes need autophagy even under basal status to maintaining homeostasis in the cells.^3, 4^ Therefore, the inhibition of autophagy–lysosomal degradation pathway is likely to have an essential role in the progression of DN.^5, 6^

Autophagy is responsible for the removal of superfluous or damaged organelles, which is essential for the survival, differentiation, development and homeostasis of cells.^7^ Previous researches have indicated that autophagy dysfunction was associated with podocyte injuries and massive proteinuria in diabetic patients.^8, 9^ Therefore, many works have focused on the responsive mechanisms about autophagy dysfunction in podocytes.^10, 11^ The classic autophagy pathways such as Atg5, Atg7, mTOR and LC3 were revealed to be involved in the autophagy dysfunction in podocytes.^12, 13^ And high-fat diet and blood glucose were considered to be the initial factors for the autophagy dysfunction in podocytes.^3, 6^ However, it is still remain unclear about what might be the trigger for the autophagy dysfunction in podocytes in DN, which might be the key to promote progression of DN.

As obesity is the primary risk factor for type 2 diabetes mellitus,^14^ adiopokine, like apelin, had been showed to be key factors promoting progression of DN in type 2 diabetes mellitus.^15, 16, 17^ Previous studies found that apelin aggravated the albuminuria by increasing the permeability of podocytes and endothelial cells, and the podocyte injuries were mediated by apelin triggered the ER stress.^16^ Apelin had been reported to inhibit autophagy in cells through activation of PI3K/Akt/mTOR pathways.^18, 19^ In this study, it is hypothesized that apelin promoted the progression of DN by inhibiting autophagy in podocytes, which resulted in podocyte apoptosis and massive proteinuria in DN.

## Results

### Apelin deteriorates renal function in diabetic mice

**Figure 1:**
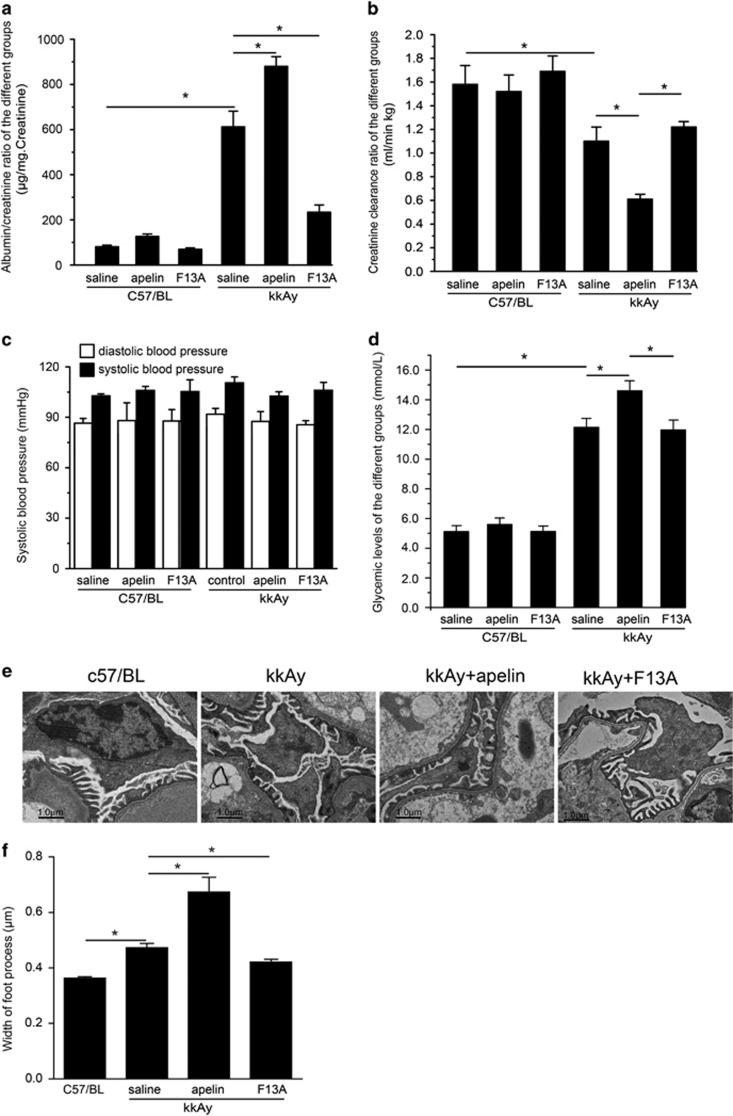
Effects of apelin on renal function, blood pressure and blood glucose concentration. (**a**) kkAy mice showed higher albuminuria compared to C57/BL mice. Apelin treatment for 4 weeks increased albuminuria in kkAy mice and F13A decreased albuminuria in kkAy mice. (**b**) KkAy mice showed decreased Ccr. Apelin treatment for 4 weeks further decreased Ccr while F13A treatment did not show any effects on Ccr. (**c**) Systolic and diastolic blood pressure did not show any difference between groups. (**d**) kkAy mice showed high blood glucose concentration. Apelin treatment for 4 weeks increased blood concentration in kkAy mice while F13A did not show any effects on glycemic levels. (**e**) Representative figures of foot process by electronic microscope. (**f**) Quantitative data for width of foot process. Apelin increased the width of foot process in kkAy mice while F13A decreased it. *n*=10, *P<0.05

The effects of apelin on renal dysfunction were examined in mice. At the age of 12 weeks old, C57/BL and kkAy mice showed different urinary albumin creatinine ratio (ACR, 76.0±4.6 and 382.3±25.1 *μ*g/mg, *P*<0.05) and creatinine clearance ratio (Ccr, 1.71±0.15 and 1.13±0.13 ml/min/kg, *P*>0.05; *n*=30, data not show). Four weeks later, the ACR was increased to 81.2±6.6 *μ*g/mg in C57/BL mice and 612 ±69.4 *μ*g/mg in kkAy mice; the Ccr was decreased to 1.58±0.16 ml/min/kg in C57/BL mice and 1.10±0.12 ml/min/kg in kkAy mice. Apelin, at the dose of 30 *μ*g/kg/day, increased ACR to 126.8±10.9 *μ*g/mg in C57/BL mice and 879.3±43.2 *μ*g/mg in kkAy mice (*n*=10, *P*<0.05). F13A (the antagonist of apelin), at the dose of 25 *μ*g/kg/day, decreased ACR to 69.7±6.6 *μ*g/mg in C57/BL mice and 234.3±31.6 *μ*g/mg in kkAy mice (*n*=10, *P*<0.05; Figure 1a). Apelin, at the dose of 30 *μ*g/kg/day, decreased Ccr to 1.52±0.14 ml/min/kg in C57/BL mice and 0.61±0.04 ml/min/kg in kkAy mice (*n*=10, *P*<0.05); F13A (the antagonist of apelin), at the dose of 25 *μ*g/kg/day, increased Ccr to 1.69±0.13 ml/min/kg in C57/BL mice and 1.22±0.05 ml/min/kg in kkAy mice (*n*=10, *P*>0.05; Figure 1b). These results indicated that increased apelin might contribute to renal dysfunction and aggravate the progression of DN.

To exclude the effects of blood pressure on renal function, systolic and diastolic blood pressure were detected in every group. No significance was observed in C57/BL, kkAy mice, and apelin- (at the dose of 30 *μ*g/kg/day) or F13A (the antagonist of apelin, at the dose of 25 *μ*g/kg/day)-treated mice (*n*=10; Figure 1c). These results suggested that apelin or its antagonist F13A had effects on progression of DN independent of blood pressure at the dose used in this study.

Because of the effects of blood glucose on renal function, the study detected the blood glucose in every group. The results indicated that apelin (at the dose of 30 *μ*g/kg/day) did increase the blood glucose concentration in C57/BL (from 5.23±0.46 to 5.78±0.56 mmol/l, *P*>0.05) and in kkAy mice (from 12.49±0.56 to 14.86±0.71 mmol/l, *P*<0.05) while F13A (the antagonist of apelin, at the dose of 25 *μ*g/kg/day) decreased the blood glucose concentration in kkAy mice (from 12.49±0.56 to 11.34±0.52 mmol/l, *P*>0.05, *n*=10; Figure 1d). These results suggested that apelin promoted the renal dysfunction in DN partly through increasing blood glucose.

As foot process is important for podocyte function, the study detected the foot process in glomeruli with electronic microscope. The results indicated that foot process was fused or effaced in kkAy mice, which was aggravated by apelin (at the dose of 30 *μ*g/kg/day) and reversed by F13A (the antagonist of apelin, at the dose of 25 *μ*g/kg/day) as shown in Figures 1e and f. These results were consistent with previous reports.^16, 20^

### Renal function was negatively correlated with podocyte apoptosis and foot process injuries

**Figure 2:**
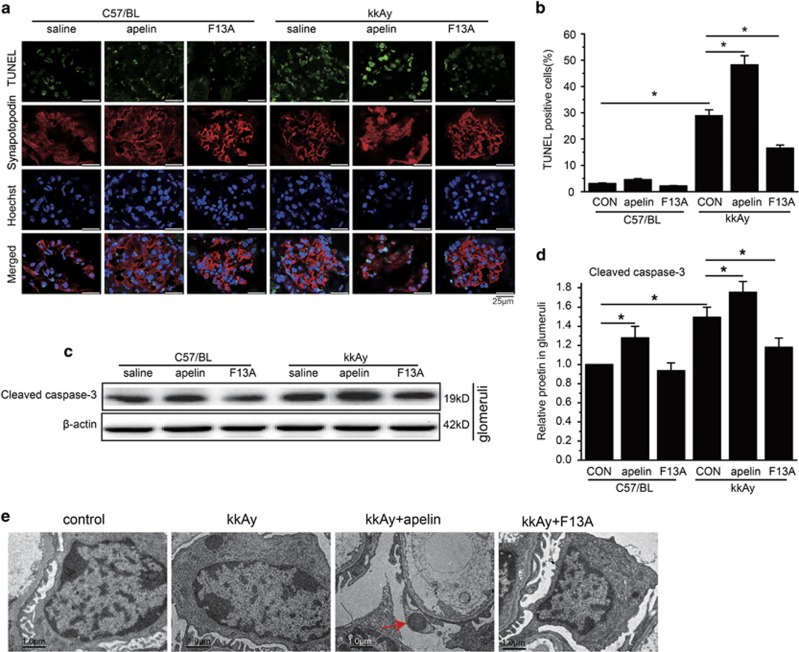
Effects of apelin on podocyte apoptosis. (**a**) Representative image of TUNEL and synapotopodin staining in C57/BL and kkAy mice with or without apelin treatment. (**b**) The number of TUNEL-positive cells in each glomerulus. (**c**) Representative images of western blot for cleaved caspase-3 in glomeruli. (**d**) Relative expression of cleaved caspase-3 in glomeruli. (**e**) Representative images for electronic microscope detecting apoptosis in C57/BL and kkAy mice with or without apelin or F13A treatment. *n*=3, *P<0.05

To observe the effects of apelin on podocyte apoptosis, terminal deoxynucleotidyltransferase-mediated DUTP-biotin nick end labeling (TUNEL) and synapotopodin were stained in the kidney. The results indicated that TUNEL- and synapotopodin-positive cells were increased to 28.9±2.2% in kkAy mice (*P*<0.05 *versus* C57/BL mice), apelin (the antagonist of apelin, at the dose of 30 *μ*g/kg/day) increased it to 48.2±3.5% in kkAy mice (*P*<0.05 *versus* kkAy mice) while F13A (the antagonist of apelin, at the dose of 25 *μ*g/kg/day) decreased it to 16.6±1.1% in kkAy mice (*P*<0.05 *versus* kkAy mice; *n*=10; Figures 2a and b).

Caspase-3 in glomeruli was detected with western blot. The results indicated that caspase-3 was increased to 145.0±9.0% in glomeruli of kkAy mice compared to that of C57/BL mice (*P*<0.05 *versus* C57/BL), which was enhanced by apelin (at the dose of 30 *μ*g/kg/day) to 176.5±10.0% (*P*<0.05 *versus* kkAy mice) and reversed by F13A (the antagonist of apelin, at the dose of 25 *μ*g/kg/day) to 121.3±8.0% (*P*<0.05 *versus* kkAy mice; *n*=3; Figures 2c and d). These results suggested that apelin may promote the progression of DN by inducing podocyte apoptosis.

To confirm the apoptosis in podocytes, electronic microscope was used to observe the change of apoptosis in podocytes. The results indicated that apoptotic nucleus was occasionally observed in kkAy mice; apelin (at the dose of 30 *μ*g/kg/day) increased the apoptotic nucleus while F13A (the antagonist of apelin, at the dose of 25 *μ*g/kg/day) decreased the apoptotic nucleus of podocytes in diabetic mice as shown in Figure 2e. These results suggested that apelin might aggravate DN by increasing apoptosis in podocytes.

### Podocyte apoptosis was negatively correlated with podocyte autophagy

**Figure 3:**
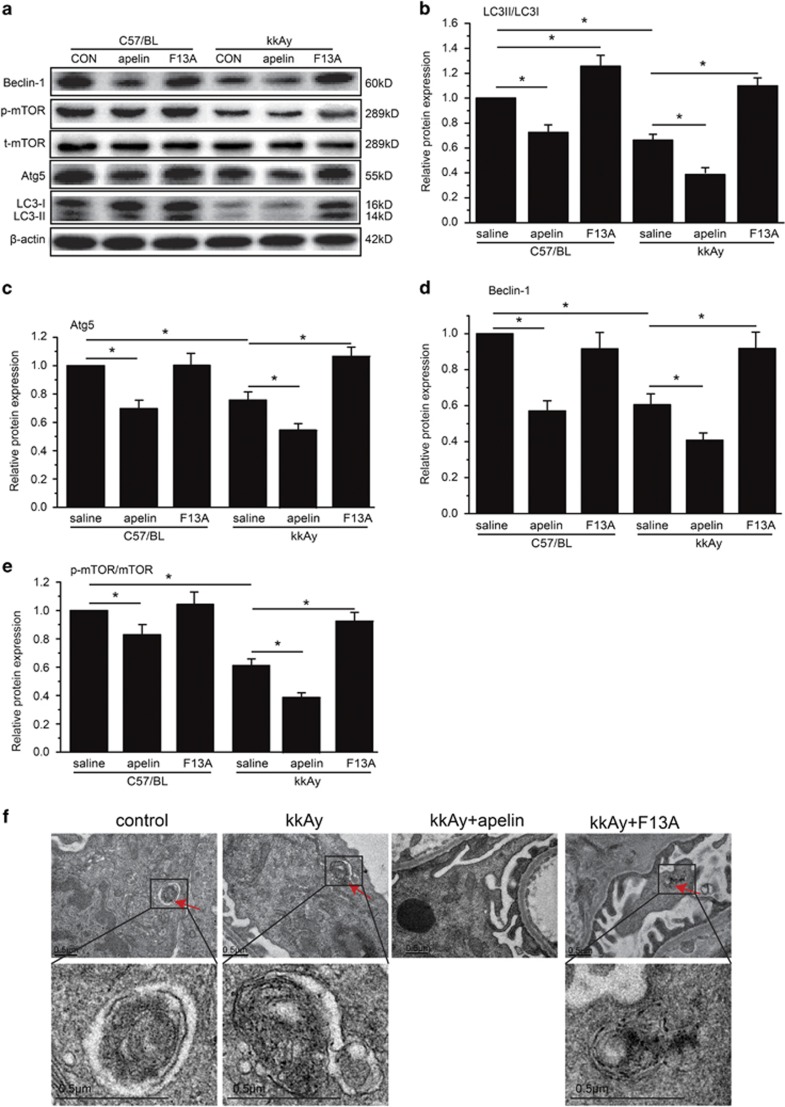
Effects of apelin on autophagy in C57/BL and kkAy mice. (**a**) Representative images of western blot for proteins mediating autophagy in glomeruli. (**b**) Relative expression of LC3II/LC3I in glomeruli with or without apelin or F13A treatment. (**c**). Relative expression of Atg5/*β*-actin in glomeruli with or without apelin or F13A treatment. (**d**) Relative expression of Beclin-1/*β*-actin in glomeruli with or without apelin or F13A treatment. (**e**) Relative expression of p-mTOR/mTOR in glomeruli with or without apelin or F13A treatment. (**f**) Representative images for electronic microscope detecting autophagsome in C57/BL and kkAy mice with or without apelin or F13A treatment. *n*=3, *P<0.05

It was reported that podocyte apoptosis might be due to inhibition of cell autophagy.^6^ Therefore, podocyte autophagy pathways were detected with western blot for the glomeruli in diabetic mice. LC3, Beclin-1 and Atg5 were detected to be significantly decreased in kkAy mice compared to C57BL mice (*n*=3, *P*<0.05; Figures 3a–d). Apelin (at the dose of 30 *μ*g/kg/day) decreased these autophagy-associated proteins to 40.1±2.7, 43.2±4.3 and 55.2±5.1% in kkAy mice (*P*<0.05 *versus* kkAy mice) while F13A (the antagonist of apelin, at the dose of 25 *μ*g/kg/day) increased these to 110.8±4.8, 93.1±9.1 and 108.2±9.1% (*P*<0.05 *versus* kkAy mice; *n*=3; Figures 3a–d). These results suggested that apelin might inhibit cell autophagy in diabetic mice.

To confirm that autophagy was in podocytes of glomeruli, electronic microscope was used to observe the autophagsome in podocytes. The results showed that autophagy was decreased in saline- and apelin-treated kkAy mice while F13A-, the antagonist of apelin, treated kkAy mice and controlled C57/BL mice displayed more autophagy in podocytes (Figure 3f).

**Figure 4:**
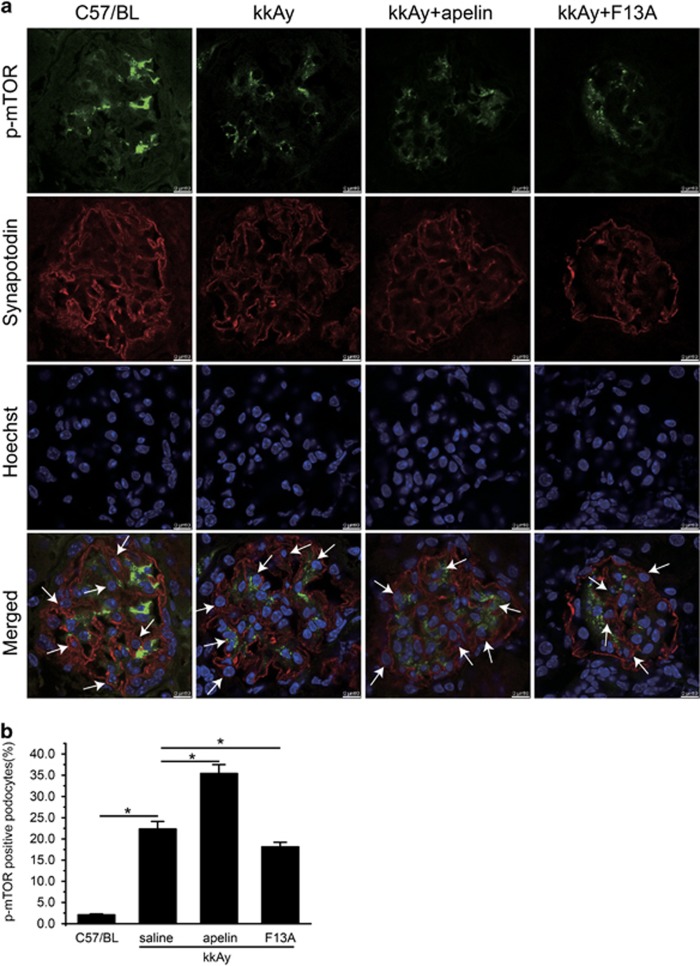
Effects of apelin on p-mOTR in podocytes. (**a**) Representative images of colocalization of p-mTOR and synaptodin in glomeruli. P-mTOR (green); synapotodin (red). (**b**) Percentage of p-mTOR positive cells in podocytes (synaptodin positive cells). *n*=3, *P<0.05

However, p-mTOR seemed like contradictory to the inhibitory effects of apelin on autophagy. As shown in Figure 3e, p-mTOR was significantly decreased in kkAy mice; apelin (at the dose of 30 *μ*g/kg/day) decreased p-mTOR to 39.8±2.9% while F13A (the antagonist of apelin, at the dose of 25 *μ*g/kg/day) increased it to 96.8±3.2% (*P*<0.05 *versus* kkAy mice). Therefore, p-mTOR and synapotodin staining was performed to evaluate p-mTOR in podocytes. The results indicated that p-mTOR in podocytes was significantly increased from 22.3±1.8 to 35.4±2.1% by apelin and decreased to 18.1±1.1% by F13A in kkAy mice (*n*=3, *P*<0.05; Figure 4).

**Figure 5:**
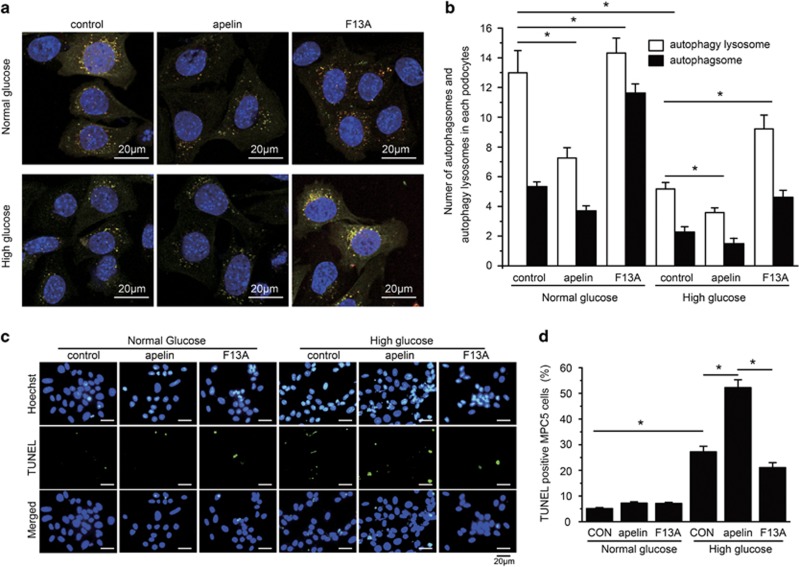
Effects of apelin on podocyte (MPC5 cells) autophagy and apoptosis. (**a**) Representative images for autophagy lysosomes and autophagsomes in MPC5 cells detected with GFP-RFP-LC3 virus. (**b**) The number of autophagsomes and autophagy lysosomes in each cultured MPC5 cells treated with NG, HG concentrations, apelin or F13A. (**c**) Representative images for TUNEL staining in MPC5 cells treated with NG, HG concentrations, apelin or F13A. (**d**) The percentage of TUNEL-positive MPC5 cells. *n*=3, *P<0.05

To test the effects of apelin on podocyte autophagy, MPC5 cells were used to detect the autophagy stream with LC3-RFP-GFP adenovirus. The results indicated that high glucose (HG) decreased autophagosomes (5.3/cell in normal glucose; NG) and autophagy lysosomes (13.0/cell in NG) in cultured MPC5 cells to 2.6/cell and 5.5/cell (*n*=3, *P*<0.05; Figures 5a and b), and apelin enhanced these to 1.9/cell and 3.6/cell while its antagonist, F13A reversed the autophagosomes and autophagy lysosomes to 4.2/cell and 8.9/cell, respectively (*n*=3, *P*<0.05; Figures 5a and b). These results suggest that apelin might enhance HG-induced podocyte injuries by inhibiting podocyte autophagy.

To test the effects of apelin on podocyte apoptosis, MPC5 cells were used to detect TUNEL with cell staining. The results indicated that HG increased TUNEL-positive cells from 5.1±0.41 to 27.2±2.2% (*n*=3, *P*<0.05; Figures 5c and d), and apelin enhanced TUNEL-positive cells to 52.2±3.1% while its antagonist F13A reversed it to 21.1±1.9% (*n*=3, *P*<0.05; Figures 5c and d).

**Figure 6:**
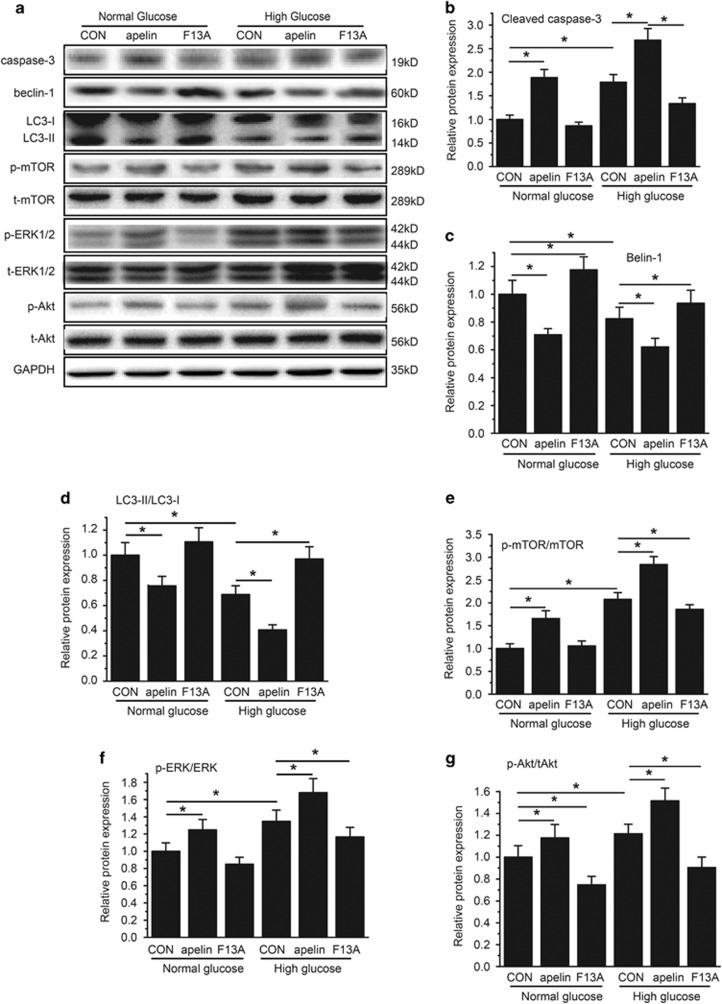
Detection of cell signaling pathways mediating podocyte autophagy and apoptosis in cultured native podocytes. (**a**) Representative images for western blot of cell signaling proteins. (**b**) Relative expression of cleaved caspase-3 in cultured native podocytes in every group. (**c**) Relative expression of Beclin-1 in cultured native podocytes in every group. (**d**) Relative expression of LC3II/LC3I in cultured native podocytes in every group. (**e**) Relative expression of p-mTOR/mTOR in cultured native podocytes in every group. (**f**) Relative expression of p-ERK/ERK in cultured native podocytes in every group. (**g**) Relative expression of p-Akt/Akt in cultured native podocytes in every group. *n*=3, *P<0.05

To test the effects of apelin on podocyte apoptosis, native cultured podocytes were used to detect apoptotic pathway with western blot. The results indicated that HG (25 mmol/l d-glucose) increased cleaved caspase-3 to 172.3±11.0%, and apelin (1.0 nmol/l) enhanced cleaved caspase-3 to 272.2±14.0% while its antagonist F13A (1.0 nmol/l) decreased cleaved caspase-3 to 127.5±4.2% in HG (25 mmol/l d-glucose)-treated podocytes (*n*=3, *P*<0.05; Figures 6a and b). On the other hand, Beclin-1 and LC3II/LC3I showed the opposite trend with caspase-3 as shown in Figures 6a, c and d. These results indicated that apelin aggravated the HG-induced podocyte apoptosis, which is negatively correlated to podocyte autophagy.

### Apelin inhibits podocyte autophagy through mTOR pathway

To investigate the cell signaling pathways for apelin to inhibit podocyte autophagy, mTOR pathways were detected with western blot in native cultured podocytes. The results showed that p-mTOR/mTOR was increased by HG (25 mmol/l d-glucose) in cultured native podocytes. Apelin (1.0 nmol/l) enhanced while its antagonist F13A (1.0 nmol/l) reversed phosphorylation of mTOR induced by HG (25 mmol/l d-glucose) in cultured native podocytes (*n*=3, *P*<0.05; Figures 6a and e).

It is reported that mTOR could be activated by MAPK/ERK1/2 and pI3k/Akt pathways,^11^ therefore ERK1/2 and Akt were detected with western blot. The results showed that phosphorylation of ERK1/2 and Akt was induced by HG (25 mmol/l d-glucose) in cultured native podocytes. Apelin (1.0 nmol/l) enhanced while its antagonist F13A (1.0 nmol/l) reversed phosphorylation of these molecules induced by HG (*n*=3, *P*<0.05; Figures 6a, f and g).

**Figure 7:**
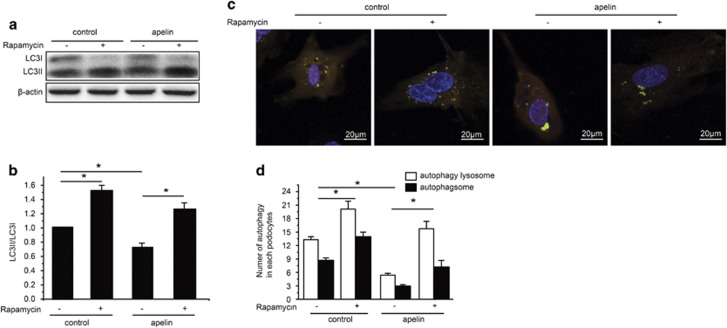
Effects of rapamycin on apelin-inhibited autophagy in cultured native podocytes. (**a**) Representative images for LC3 detection with western blot. (**b**) Relative expression of LC3II/LC3I in cultured native podocytes treated with apelin and rapamycin. (**c**) Representative images for autophagy lysosomes and autophagsomes in cultured native podocytes with apelin and rapamycin treatment detected with GFP-RFP-LC3 virus. (**d**) The number of autophagsomes and autophagy lysosomes in cultured native podocytes treated with apelin and rapamycin. *n*=3, *P<0.05

To confirm the function of mTOR pathway in apelin preventing podocyte autophagy, rapamycin was used to inhibit mTOR before and after apelin treatment in native cultured podocytes. The results showed that rapamycin increased LC3II, which was decreased by apelin in cultured native podocytes (*n*=3, *P*<0.05; Figures 7a and b). The autophagosome and autophagy lysosomes were increased by rapamycin in native cultured podocytes, which were reversed by apelin as well (*n*=3, *P*<0.05; Figures 7c and d). These results suggested that apelin activates mTOR pathway to inhibit podocyte autophagy in DN.

## Discussion

Previously, autophagy had been considered to be crucial in maintaining podocyte homeostasis,^4^ which was altered in diabetic conditions.^5^ Apelin had been reported to inhibit cell autophagy as a pro-survival mechanism,^21^ the effects of apelin on podocyte autophagy are not sufficiently addressed.^18, 21^ In this study, it is revealed that apelin promoted proteinuria in DN by inhibiting podocyte autophagy both *in vivo* and *in vitro*. Consequently, decreased autophagy enhanced apoptosis in podocytes, leading to damage of glomerular filtration membrane. Apelin-treated kkAy mice therefore showed increased proteinuria, podocyte apoptosis and foot process effacement as shown in Figures 1 and 2, which might contribute to progression of DN. These results are consistent with suggestions from other investigators that apelin inhibiting autophagy^18^ and autophagy deficiency is involved in the pathogenesis of DN.^3^

High levels of constitutive autophagy have been demonstrated in podocytes under basal conditions, and podocyte-specific deletion of Atg5 resulted in podocyte loss and late-onset glomerulosclerosis in aging mice,^22^ highlighting the importance of autophagy in the maintenance of the function and integrity of podocytes. However, in certain settings, uncontrolled massive autophagy may lead to cell death.^23^ This study provided the evidence that apelin inhibited autophagy in podocytes to enhance proteinuria in DN.

First of all, the data revealed that increased proteinuria and decreased Ccr were related to apelin-induced podocyte apoptosis and foot process effacement in DN as shown in Figures 1 and 2. The pro-apoptotic effect of apelin on podocytes was confirmed *in vitro* as shown in Figures 5c and d: HG increased apoptosis in MPC5 cells; apelin increased apoptosis in HG-treated podocytes but did not show such effects on NG-treated podocytes. These results suggested that apelin might display synergistic effect with HG to promote apoptosis of podocytes in diabetic conditions. However, previous studies indicated that apelin prevent apoptosis in cardiomyocytes and other cells,^24, 25^ which is paradox with the results in podocytes. Therefore, the difference between cardiomyocytes and podocytes might be the key to lead to these results. Both cardiomyocytes and podocytes are terminal differentiated cells, however, podocyte is dependent on autophagy to sustain homeostasis in the cell while cardiomyocytes are not.^4, 26^ Previous reports have showed that apelin protected heart from injury by preventing autophagy of cardiomyocytes,^27^ therefore the effects of apelin on podocyte autophagy might be the key points that mediated the promoting effects of apelin on DN.

To confirm that apelin promotes apoptosis by inhibiting autophagy in podocyte, podocyte autophagy was observed both *in vivo* and *in vitro*. KkAy mice showed considerable attenuation in podocyte autophagy when treated with apelin and enhancement of that when treated with F13A (antagonist of apelin) as shown in Figure 3f. Apelin decreased autophagy in HG-treated MPC5 cells while F13A (antagonist of apelin) increased it as shown in Figures 5a and b. These results suggest that apelin might induce podocyte apoptosis through preventing podocyte autophagy. Then, what is the cell signaling pathway for apelin preventing podocyte autophagy?

Recently, apelin has been reported to ameliorate autophagy through mTOR pathway.^12^ Hence, the phosphorylation of mTOR was observed both in glomeruli and in cultured native podocytes. Apelin decreased phosphorylation of mTOR in glomeruli of kidney (as shown in Figure 3) while F13A (antagonist of apelin) increased phosphorylation of mTOR. These results suggested that apelin may increase autophagy in glomeruli. However, the results in cultured native podocytes were different: apelin increased phosphorylation of mTOR while F13A (antagonist of apelin) decreased phosphorylation of mTOR. These results suggested that apelin inhibited autophagy in podocytes by increasing phosphorylation of mTOR. The conflicting results between glomeruli and cultured native podocytes might be due to the different cell components. There are many kinds of cells in glomeruli, such as endothelial cells, mesangial cells and so on, which might show different trends in apelin-regulated phosphorylation of mTOR. This issue was confirmed by p-mTOR and synapotodin staining. As shown in Figure 4, p-mTOR-positive podocytes were increased by apelin treatment and decreased by F13A (antagonist of apelin). Therefore, apelin might inhibit autophagy through increasing phosphorylation of mTOR in podocytes.

To confirm the cell signaling pathway of apelin on autophagy in podocytes, Akt and ERK1/2 were observed. Apelin enhanced HG-induced phosphorylation of both Akt and ERK1/2 while F13A (antagonist of apelin) reversed these effects in HG-treated podocytes. At the same time, LC3II, Beclin-1 and caspase-3 were observed to analyze the autophagy and apoptosis of the cultured cells. The results indicated that both HG and apelin decreased LC3II and Beclin-1, and increased caspase-3, while its antagonist F13A showed the opposite effects. These results indicated that HG and apelin might prevent podocyte autophagy through Akt and ERK1/2 pathway.

To confirm that apelin prevent podocyte autophagy through mTOR pathway, LC3, autophagosome and autophagy lysosome were observed after rapamycin was used to inhibit mTOR. The results showed that the increased LC3II, autophagosome and autophagy lysosome by rapamycin treatment were reversed by apelin as shown in Figure 7. These results suggested that apelin inhibited autophagy in podocytes through activating mTOR pathway.

In conclusion, this study indicated that increased apelin concentration in diabetic patients promoted the progression of DN by inhibiting autophagy in podocytes through ERK, Akt and mTOR pathway. Dysfunction of autophagy in podocytes will lead to organelle disorder, such as ER stress as previously reported,^16^ which induced foot process effacement and podocyte apoptosis and lead to the increased permeability of the filtration membrane in glomeruli, proteinuria and renal dysfunction in DN.

## Materials and methods

### Ethics statement

All animal studies followed the Animal Care and Use Committee of Capital Medical University (20100610). All animals received humane care, and the experimental protocol was approved by the Committee of Laboratory Animals according to the institutional guidelines.

### Animal model

Because of the marked obesity, glucose intolerance, severe insulin resistance, dyslipidemia and hypertension displayed in the mice, kkAy mouse has been used as a polygenic model for human type 2 diabetes mellitus.^17^

Male kkAy mice and control C57BL/6 J mice at 12 weeks of age were purchased from Capital Medical University (Beijing, China). Mice were fed on semi-purified moderately high-fat diet containing 24% kcal fat and 0.2% cholesterol. Mice were randomized according to albumin/creatinine at 12 weeks of age and were killed at 16 weeks of age.

C57BL/6 J mice were classified as normal control (C57/BL,*n*=10), and kkAy mice were considered DN when their urine ACR was ⩾300 *μ*g/mg. The mice were then randomly divided into DN group (kkAy group, *n*=10), which were intraperitoneally injected with vehicle; apelin treatment DN group (kkAy+apelin, *n*=10), which were intraperitoneally injected with apelin-13 (A6469; Sigma-Aldrich, St. Louis, MO, USA, 30 *μ*g/kg/day) for 4 weeks; and F13A treatment group (kkAy+F13A, *n*=10), which were intraperitoneally injected with F13A (the antagonist of apelin-13,057-29; Phoenix Pharmaceuticals, Strasbourg, France, 25 *μ*g/kg/day) for 4 weeks. Systolic and diastolic blood pressures were measured by the tail-cuff system (Softron BP-98 A; Softron, Tokyo, Japan) at the end of the experiments.

### Biochemical characterization

The level of fasting serum creatinine and fasting bodyweight were measured at the end of the experiments. The urine samples were collected for a period of 24 h using a mouse metabolic cage (CLEA, Tokyo, Japan). Urinary albumin and creatinine were measured by immunoassay (DCA 2000 system; Siemens AG, Munich, Germany). All analyses were performed in accordance with the manuals provided by the manufacturers. The urinary ACR=urinary albumin (*μ*g)/urinary creatinine (mg). Ccr=(urinary creatinine (mg/dl)/urinary volume (ml)/serum creatinine (mg/dl))/(1000/bodyweight (g))/(1/1440 (min)).

### Immunostaining

Kidneys were excised carefully without any damage and embedded in OCT (4583, SAKURA Tissue-Tek&reg, Torrance, CA, USA) on dry ice. Sections of 5 *μ*m were cut and performed with TUNEL (G3250; Promega Corp., Madision, WI, USA) staining. After fixed in 10% neutral buffered formalin, the slices were washed with PBS and treated with 0.2% Triton X-100, equilibrated with equilibration buffer for 10 min and incubated in rTdT Incubation Buffer for 60 min at 37 °C, then in 2 × SSC for 15 min at room temperature and incubated with Hoechst 33342 (1:20 000) for 5 min. The slices were fixed for another time and incubated with rabbit anti synapotodin (sc-50459; Santa Cruz Biotechnology, Santa Cruz, CA, USA) and donkey anti-rabbit-Alex647 (ab150075; Abcam, Cambridge, UK). Images were obtained using a confocal microscope (TCS-SP8; Leica, Buffalo Grove, IL, USA). Positive cells with both synapotodin and TUNEL located in the glomeruli were counted and divided by synapotodin-positive cells located in the glomeruli. At least 100 glomeruli were analyzed in each group.

p-mTOR and synaptodin staining was performed with OCT-embedded fresh kidneys. Briefly, sections of 5 *μ*m were cut and fixed in 10% neutral buffered formalin; the slices were then washed with PBS and treated with 0.2% Triton X-100; mouse anti-p-mTOR antibody (sc-293133; Santa Cruz Biotechnology) and rabbit anti synapotopodin (sc-50459; Santa Cruz) were incubated with the slices after blocked with 1% BSA; donkey anti-mouse IgG-488 (ab150105; Abcam, Shanghai, China) and donkey anti-rabbit IgG-647 (ab150075; Abcam) were incubated with the slices; Hoechst 33342 was then used to stain nucleus; the slices were mounted in VECTASHIELD Mounting Medium (H-1000, Vector Laboratories, Inc., Burlingame, CA, USA). The images were captured with confocal microscope (TCS-SP8; Leica DM6000 CS). The percentage of p-mTOR-positive cells in synapotodin-positive cells was analyzed in each glomerulus.

### Analysis for electronic microscope

A saliquot of kidney cortical tissue was cut into 1 mm^3^ pieces and fixed in 2.5% glutaraldehyde in Millonig solution and embedded in PolyBed 812 (08792, Polysciences Inc., Warrington, PA, USA) for electronic microscope analysis. The images were obtained from transmission electron microscope (JEM-1400plus, JEOL Ltd., Akishima, Tokyo, Japan). The width of the foot process was analyzed with ImageJ software (NIH, Bethesda, MD, USA).

### Podocyte cell culture

Conditionally immortalized mouse podocytes, kindly provided by P Mundel (Mt. Sinai School of Medicine, New York, NY, USA), were cultured as previously described.^28^ When podocytes were well-differentiated, they were serum-starved overnight with serum-free RPMI 1640 medium with NG (5.5 mmol/l d-glucose) or HG (25 mmol/l d-glucose) with or without apelin (1.0 nmol/l) or F13A (1.0 nmol/l) modulation for 1–48 h and collected for the following assays. All the experiments were repeated for at least three times.

Native podocytes were isolated from kidney of mice. Briefly, glomeruli were prepared by filtration of the cortex of kidney with mesh sieves, which were100, 76 and 54 *μ*m in size, the tissues left on the mesh sieve with 54*μ*m in size were collected and transferred to cultural plates and incubated with 20% serum in mixture of DMEM and RPMI 1640 for 5 days. The cells were identified with synapotodin staining as shown in Supplementary Figure 1. Other cells left were used for the following experiments. After starvation with serum-free DMEM, the cells were modulated with apelin (1.0 nmol/l) or F13A (1.0 nmol/l) and/or treated with NG (5.5 mmol/l d-glucose) or HG (25 mmol/l d-glucose) for 1–48 h. The cells were then collected for the following assays. All the experiments were repeated for at least three times.

### Assay for podocyte injuries

RFP-GFP-LC3 adenovirus was used to detect the autophagy in podocytes. LC3 expressed by this virus permits the distinction between acidic and nonacidic LC3-positive structures, because GFP is acid-sensitive, whereas RFP is not. Total LC3-positive dots are GFP+/RFP+ and GFP−/RFP+ dots, early autophagysomes are GFP+/RFP+ dots and autophagy lysosomes are GFP−/RFP+ dots. Podocytes on coverslips were infected with RFP-GFP-LC3 as described. In brief, the medium was removed and replaced for 5 h by RPMI 1640 medium followed by RFP-GFP-LC3 adenovirus infection.^29^ Confocal images were acquired after 48 h treatment as described in podocyte culture using Zeiss LSM 710 Meta confocal laser scanning microscope (73447, Oberkochen, Germany). Total LC3-positive dots, early autophagosomes and autophagy lysosomes were analyzed using ImageJ software and expressed as a percent of early autophagosomes or autophagy lysosomes in total LC3-positive dots in cells. At least 1000 cells were analyzed.

### TUNEL staining

TUNEL staining was used for the analysis of apoptosis in podocytes. A TUNEL staining kit, DeadEnd Fluorometric TUNEL System (G3250, Promega Corp., Madision, WI, USA) was used for TUNEL staining according to the manufacturer’s instruction, just as described in immunostaining. Cell counting was performed in five non-overlapping fields captured on an Olympus BX63 microscope (Shanghai, China) at × 20 magnification. The percentage of TUNEL-positive cells to total cells was counted and calculated using ImageJ software (NIH). At least 1000 podocytes each in three dishes were counted for every group.

### Western blotting of protein expression

The proteins from prepared glomeruli or cultured cells were quantified with BCA protein Assay kit (23228; Thermo Scientific, Rockford, IL, USA). The same mass of protein (50 *μ*g) in every group were loaded to 6–15% SDS-PAGE gel, and fractionated by electrophoresis. The protein in the gel was transferred to polyvinylidene difluoride filter membranes with electro-blot and incubated with the primary antibody at 4 °C, and then with a HRP-conjugated secondary antibody.

*Primary antibodies*: Rabbit anti-APLNR (sc-33823; Santa Cruz Biotechnology), goat anti-LC3 (12741; Cell Signaling Technology, Danvers, MA, USA), rabbit anti-Beclin-1 (sc-11427; Santa Cruz Biotechnology), rabbit anti-caspase-3 (9662s; Cell Signaling Technology), rabbit anti-mTOR (2983; Cell Signaling Technology), rabbit anti-pmTOR (5536; Cell Signaling Technology), rabbit anti-p-Akt(9271s; Cell Signaling Technology), rabbit anti-Akt (4691s; Cell Signaling Technology), rabbit anti-pERK1/2 (4370s; Cell Signaling Technology), mouse anti-ERK1/2 (4695s; Cell Signaling Technology), rabbti anti-Atg5 (2630S; Cell Signaling Technology) and rabbit anti synapotopodin (sc-50459; Santa Cruz). The secondary antibodies are donkey anti-rabbit-HRP (sc-2313; Santa Cruz Biotechnology) and donkey anti-goat-HRP (sc-2020; Santa Cruz Biotechnology).

The blotted protein on the membrane was developed with chemiluminescent HRP substrate (WBKLS0100, Millipore Corporation, Billerica, MA, USA) and captured with Fluor Chem FC3 (Protein Simple, Wallingford, CT, USA). Densitometry was performed with ImageJ software (NIH). To verify equal loading, antibody to GAPDH was used.

### Statistics

Data were summarized as mean±S.D. A *P*-value <0.05 was considered significant. All reported *P*-values are two-sided. Analysis was carried out using SPSS version 13.0 for the PC (Armonk, NY, USA). Differences between different groups were evaluated for significance using independent *t*-test or one-way ANOVA and Newman–Keuls *post hoc* tests.

## Publisher’s Note

Springer Nature remains neutral with regard to jurisdictional claims in published maps and institutional affiliations.
