# Supplementary material for: Apelin involved in progression of diabetic nephropathy by inhibiting autophagy in podocytes
Source: Cell Death Dis. 2017 Aug 24;8(8):e3006–. doi: 10.1038/cddis.2017.414 (PMC5596593; doi:10.1038/cddis.2017.414)
Supplement: Supplementary Figure Legend [file cddis2017414x1.docx]

Supplementary figure 1: Identification for native cultured podocytes. Synaptotodin was stained with Hoechst, the results indicated that most of the cell was positive with synaptodin.
