# Supplementary figures and images for: Apelin involved in progression of diabetic nephropathy by inhibiting autophagy in podocytes
Source: Cell Death Dis. 2017 Aug 24;8(8):e3006–. doi: 10.1038/cddis.2017.414 (PMC5596593; doi:10.1038/cddis.2017.414)

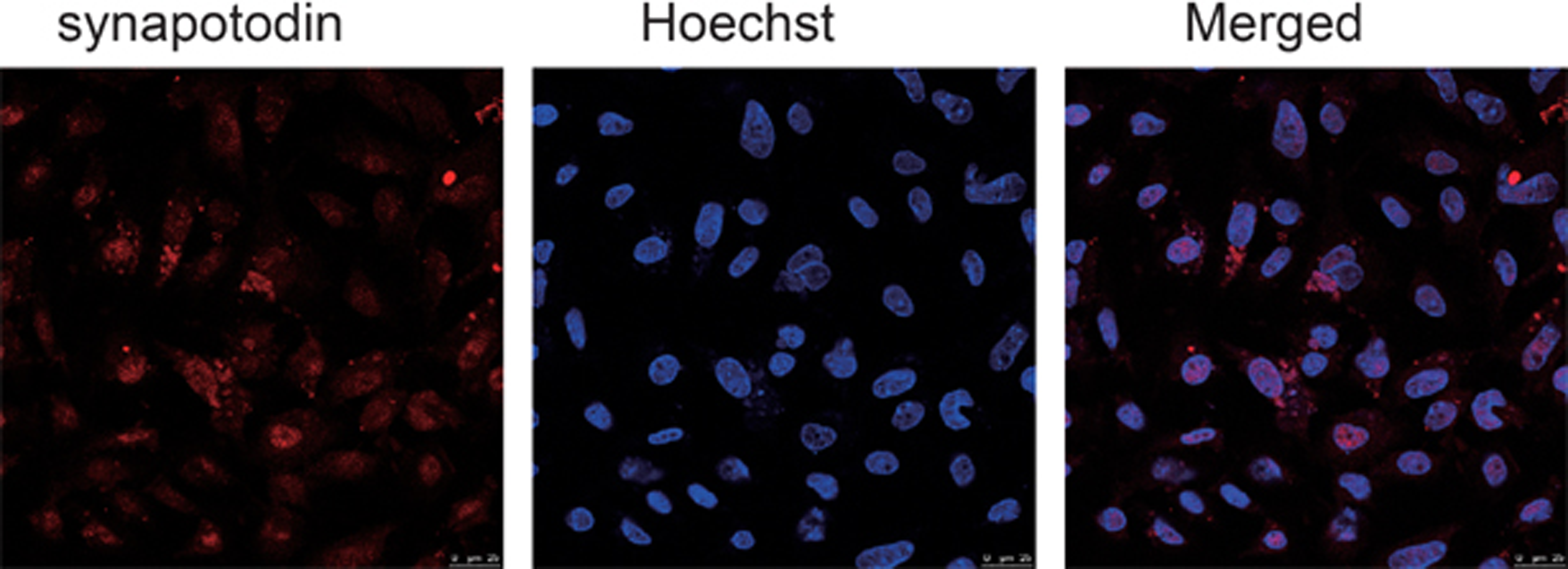

Supplement: Supplementary Figure 1 [file cddis2017414x2.tif]
